# Can stereotactic body radiotherapy replace brachytherapy in gynecologic cancers? A systematic review of current evidence

**DOI:** 10.1016/j.ctro.2026.101226

**Published:** 2026-07-17

**Authors:** Paula Vicente Ruiz, Ana Illescas Vacas, Ángel Vilches Arenas

**Affiliations:** a Department of Radiation Oncology, Virgen Macarena University Hospital, Avda. Dr. Fedriani 3, 41009 Seville, Spain; b Department of Preventive Medicine and Public Health, Faculty of Medicine, University of Seville, Av. Sanchez Pizjuan s/n, 41009 Seville, Spain

**Keywords:** Stereotactic body radiation therapy, Brachytherapy, Gynecologic cancer, Dose-Volume Histogram, Dose escalation

## Abstract

Brachytherapy (BT) is a cornerstone of curative radiotherapy for gynecologic cancers due to its ability to deliver high intratumoral doses with steep dose gradients. Limited availability and declining utilization have increased interest in stereotactic body radiotherapy (SBRT) as a non-invasive alternative. This systematic review evaluates the current evidence comparing SBRT and BT in gynecologic cancers. A systematic search of PubMed, Scopus, EMBASE, Cochrane Library, and Web of Science was conducted for studies published between 2014 and 2025. Eligible studies included dosimetric analyses, observational cohorts, and consensus statements comparing SBRT and BT. Outcomes of interest were target coverage, intratumoral dose escalation, organ-at-risk exposure, conformity and homogeneity indices, and overall survival when available. Eleven studies met the inclusion criteria, including nine dosimetric studies, one population-based cohort, and one consensus guideline, mainly focused on locally advanced cervical cancer. BT consistently achieved superior intratumoral dose escalation and higher central dose heterogeneity, while SBRT showed competitive peripheral target coverage and improved conformity in margin-free scenarios. These advantages were reduced when clinically realistic planning margins were applied. The only available survival analysis reported no significant difference in overall survival between techniques after adjustment for prognostic factors. BT remains the reference standard for dose escalation in gynecologic cancers. SBRT may be considered a selective alternative when BT is not feasible, but prospective studies with standardized planning and long-term follow-up are needed to define its clinical role.

## Highlights

• Brachytherapy remains the gold standard for dose escalation in gynecologic cancers.
• SBRT provides competitive target coverage but limited central dose escalation.
• Clinical outcome differences between SBRT and brachytherapy remain unclear.
• SBRT is a selective alternative when brachytherapy is not feasible.

## 1 Introduction

Gynecologic cancers remain a major global health concern, representing a significant proportion of cancer incidence and mortality among women [1]. Cervical cancer continues to be one of the most common malignancies worldwide, particularly affecting regions with limited access to screening and human papillomavirus vaccination [2], [3]. Endometrial cancer (EC) shows a steady rise in incidence, driven largely by demographic aging and increasing rates of obesity.

Radiotherapy has been central to the management of gynecologic malignancies for more than a century. Technological progress, including three-dimensional conformal radiotherapy (3D-CRT), intensity-modulated radiotherapy (IMRT), volumetric modulated arc therapy (VMAT), image-guided techniques and, more recently, stereotactic approaches, has considerably improved treatment precision and reduced toxicity [4], [5], [6].

Brachytherapy (BT) remains the standard of care in many clinical scenarios, particularly for locally advanced cervical cancer (LACC) and selected postoperative settings in EC [7], [8], [9], [10], [11], [12]. Its ability to deliver highly conformal, escalated doses to the tumor while sparing adjacent organs at risk (OARs) makes BT critical for achieving optimal local control and survival outcomes [7], [13], [14].

Despite its clinical effectiveness, BT presents important limitations. The technique requires specialized equipment, trained personnel, and procedural expertise, resulting in heterogeneous availability across institutions and healthcare systems [15]. In addition, BT is inherently invasive and cannot be performed in some patients due to anatomical distortion, tumor obstruction, medical comorbidities, or patient refusal [16]. These limitations have stimulated increasing interest in stereotactic body radiotherapy (SBRT) as a non-invasive alternative capable of delivering highly conformal ablative doses.

SBRT enables high-precision dose delivery in a limited number of fractions and has been explored in gynecologic oncology as a boost following pelvic external beam radiation therapy (EBRT) [17], [18], [19], [20], [21], [22], [23], [24], [25]. However, available evidence remains heterogeneous, particularly regarding long-term safety, adherence to OAR dose constraints, and the risk of severe toxicities reported in some prospective trials when SBRT is used as a direct replacement for intracavitary (IC-BT) or interstitial brachytherapy (IS-BT) [26], [27], [28], [29], [30], [31], [32]. Despite growing clinical interest and the reported decline in BT utilization in some healthcare systems, there is still no clear synthesis of dosimetric and clinical evidence directly comparing both techniques [6], [33].

Therefore, this systematic review aims to evaluate the current dosimetric and clinical evidence comparing SBRT and BT in gynecologic cancers and to clarify the effectiveness, safety profile and potential role of SBRT in contemporary radiotherapy practice.

## 2 Material and methods

### 2.1 Study design and protocol

This systematic review was conducted following the Preferred Reporting Items for Systematic Reviews and Meta-Analyses (PRISMA 2020) guidelines to evaluate dosimetric and clinical evidence comparing SBRT and BT in non-metastatic gynecologic cancers. The study focused on target coverage, dose escalation using dose-volume histogram (DVH) parameters and organ-at-risk exposure according to EMBRACE II, GEC-ESTRO, SGO and ABS guidelines [12], [14], [16], [34], [35], and also available clinical outcomes. The review protocol was not registered in PROSPERO.

### 2.2 Search strategy

A systematic literature search was performed in PubMed, Scopus, EMBASE, Cochrane Library, and Web of Science to identify relevant studies published between January 2014 and October 2025. The search strategy combined Medical Subject Headings (MeSH) and free-text keywords, including “*Genital Neoplasms, Female”, “Uterine Neoplasms”, “Uterine Cervical Neoplasms”, “Vaginal Neoplasms”, “Radiosurgery”,* and “*Brachytherapy”*. Boolean operators (AND, OR, NOT) were used to combine search terms.

### 2.3 Eligibility criteria

Studies were included if they met the following criteria:

• compared between SBRT and BT in non-metastatic gynecologic cancers
• reported dosimetric or clinical outcomes
• were original research articles (observational studies, in-silico dosimetric comparative studies or consensus guidelines)
• English or French language publications
• published between 2014 and 2025

Studies were excluded if they:

• included metastatic disease
• received previously pelvic radiation
• were case reports or conference abstracts
• focused exclusively on BT or SBRT without comparative data

### 2.4 Study selection and data extraction

Two reviewers independently screened titles and abstracts to identify eligible studies. Full-text articles were then reviewed to determine final inclusion. Discrepancies in study selection or data extraction were resolved by discussion.

**Fig. 1:**
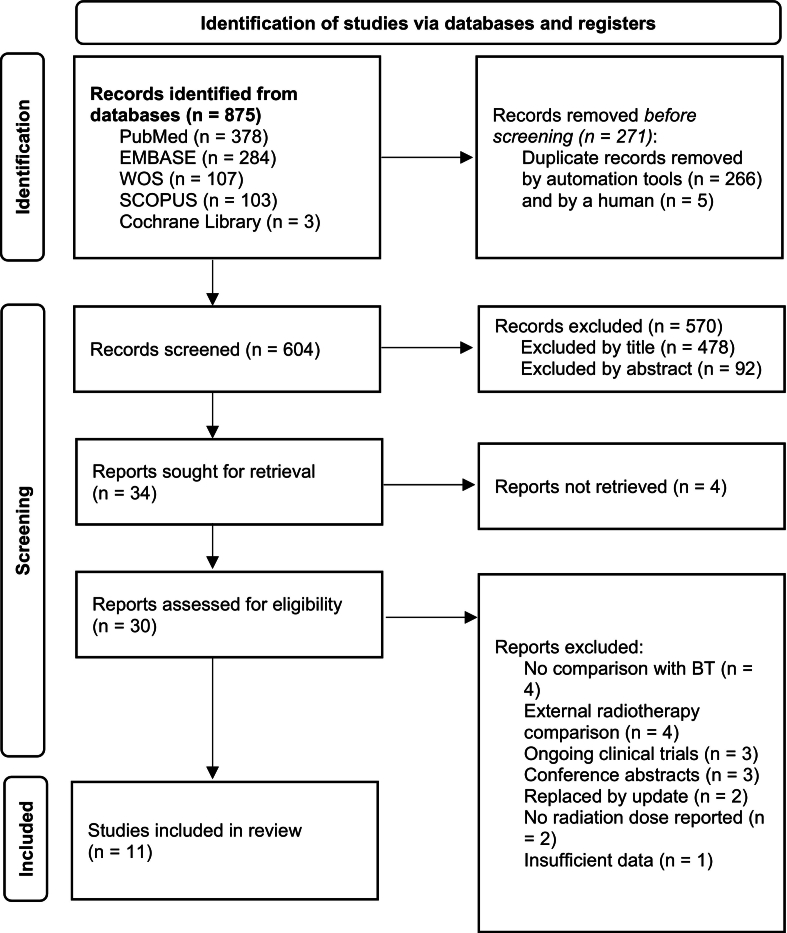
PRISMA 2020 flow diagram of study selection. Flow diagram illustrating the identification, screening, eligibility assessment, and inclusion of studies in this systematic review. The figure details the number of records identified through database searching, duplicates removed, records screened, full-text articles assessed for eligibility, reasons for exclusion, and the final number of studies included in the qualitative synthesis (*n* = 11).

The study selection process is illustrated in the PRISMA flow diagram (Fig. 1).

The following data were extracted from each study:

• first author and country

### 2.5 Study design

• cancer type
• number of patients
• treatment technique (SBRT or BT) and fraction schedule
• dosimetric parameters to target volumes: D30, D50, D90, D95, D98 (minimum dose received by at least 30%, 50%, 90%, 95% and 98% of the target volume, respectively), V100 and V150 (percentage of the target volume receiving at least 100% and 150% of the prescribed dose, respectively).
• OARs doses: dose delivered to the most irradiated 2 cm^3^ (D2cc) of the rectum (D2ccR), sigmoid colon (D2ccS), and bladder (D2ccB)
• plan quality through conformity index (CI) and the homogeneity index (HI).
• clinical outcomes when available

The extracted data are summarized in Table 1.

**Table 1:** Characteristics and main findings of included studies comparing stereotactic body radiotherapy and brachytherapy in non-metastatic gynecologic cancers. Summary of study design, patient population, conventional and alternative radiotherapy techniques, fractionation schedules, objectives and main results, including target coverage and organ-at-risk dose metrics.

Study ID Country	Design	Population	BT	SBRT	Dose fractionation	Objectives	Results (BT vs. SBRT)
O'Donnel et al. [36] United States	Retrospective observational cohort study	*n* = 15.905 LACC Post EBRT Median 45Gy/30fr	BT (*n* = 14.394)	SBRT (*n* = 42)	Median 25 Gy in 5 fractions for SBRT plan	OS univariate OS (PSM) D90 HR-CTV	99.1 vs 30.3 (p = 0.001) (*p* = 0263) N/A vs. Median 75.5
Chargari et al. [37] France	Expert consensus / clinical practice guideline	LACC	IC/IS BT (HDR)	SBRT (CK, HT, Linac, MR-Linac)		D90 HR-CTV D30/D50 HR-CTV OARs	85–90 vs. < 80 BT is superior, SBRT cannot reproduce BT hotspots SBRT often better than BT only when target under-dosed
Benkhaled et al. [38] Belgium	Dosimetric comparative study (in silico)	*n* = 17 LACC Post EBRT 45-55Gy/25fr	IC-BT vs. IC + IS-BT (HDR) MRI + CT	SBRT (VMAT, Linac) (PTV = HR-CTV)	28.4 Gy in 4 fractions	D30% HR-CTV D50% HR-CTV D90% D2cc R D2cc S D2cc B	*IC-BT* vs. *IC* *±* *IS-BT* vs. *SBRT* 14.9 vs. 15.2 vs. 10.1 (*p* < 0.001) 11.1 vs. 12.1 vs. 9.83 (*p* = 0.005 IC-BT vs SBRT, *p* < 0.001 IC + IS-BT vs SBRT) 6.62 vs. 7.8 vs. 9.02 (p < 0.001) 3.87 vs. 3.58 vs. 2.71 (p < 0.001) 3.65 vs. 3.11 vs. 2.59 (p < 0.001) 6.46 vs. 5.49 vs. 4.99 (p < 0.001)
Gao et al. [39] China	Dosimetric comparative study (in silico)	n = 20 LACC Post EBRT 48.6Gy/27	IC/IT-BT (HDR) CT	SBRT (CK) CK-CTV and CK-PTV (isotropic margin of 5 mm from HR-CTV)	28 Gy in 4 fractions	D50% D95% D98% HI CI D2cc R D2cc S D2cc B	*BT* vs. *SBRT (HR-CTV)* vs. *SBRT (PTV)* 42.08 vs. 32.49 vs. 32.53 *(p* *<* *0.001)* 25.89 vs 26.99 vs. 27.05 *(p* *<* *0.001)* 23.74 vs. 25.49 vs. 25.75 *(p* *<* *0.001)* 4.34 vs. 1.47 vs. 1.53 *(p* *<* *0.001)* 0.75 vs. 1.12 vs. 1.06 *(p* *<* *0.001)* 17.36 vs. 15.84 vs. 21.92 *(p* *=* *0019 BT* vs *CTV, p* *<* *0.001 BT* vs *PTV)* 13.44 vs. 13.98 vs. 20 *(p* *=* *1 BT* vs *CTV, p* *<* *0.001 BT* vs *PTV)* 19.64 vs. 18.54 vs. 22.48 *(p* *=* *0.007 BT* vs. *CTV, p* *<* *0.001 BT* vs *PTV)*
Otahal et al. [40] Czech Republic	Dosimetric comparative study (in silico)	*n* = 10 LACC Post EBRT 45Gy/25fr	IC-BT (*n* = 7) and IC + IT-BT (*n* = 3) HDR MRI	SBRT CK (PTV = HR-CTV)	30 Gy in 5 fractions	D30 HR-CTV D90 HR-CTV D90 GTV D2cc R D2cc S D2cc B	17.9 vs. 9.4 6.1 vs. 7.3 9.8 vs. 8.9 3 vs. 1.8 *(p* *=* *0.0007)* 3.2 vs. 2 *(p* *=* *0.008)* 4.4 vs. 3.3 *(p* *=* *0.003)*
Dahbi et al. [41] Morocco	Dosimetric comparative study (in silico)	*n* = 20 LACC Post EBRT 46Gy/23fr	IC-BT (HDR) MRI + CT	SBRT (Linac) (PTV = HR-CTV)	21 Gy in 3 fractions	D90 HR-CTV CI D2cc R D2cc S D2cc B	15 vs. 8.32 (*p* = 0.028) 0.76 vs. 0.86 (*p* = 0.671) 71.8 vs. 74.7 (*p* = 0.256) 67.9 vs. 75.75 (*p* = 0.0173) 74.6 vs. 84.7 (*p* = 0.019)
Dicer et al. [42] Czech Republic	Dosimetric comparative study (in silico)	n = 4 LACC Post EBRT 50.4-45Gy/25–28fr	IC-BT (HDR) MRI + CT	SBRT MR-Guided Adaptive Radiation Therapy (SMART) PTV (isotropic margin of 3 mm from HR-CTV)	28 Gy in 4 fractions	D90 HR-CTV D90 HR-PTV D2cc R D2cc S D2cc B	89.7 vs. 95.4 89.7 vs. 82.9 70.7 vs 70.8 65.7 vs. 73.6 86.4 vs. 81.4
Neumann et al. [43] Germany	Dosimetric comparative study (in silico)	n = 11 LACC Post EBRT 50.5Gy/28fr	IC-BT HDR MRI + CT Compared with BTRRS and BTref	RRS CK Compared with RRS70 and RRS25 (PTV = HR-CTV)	30 Gy in 5 fractions	V100% HR-CTV D90 HR-CTV CN D2cc R D2cc S D2cc B	*BTRRS* vs. *BTref* vs. *RRS70* vs. *RRS25* 80.6 vs. 90.9 vs. 97.1 vs. 100 25.7 vs. 32 vs. 31.8 vs. 69.5 0.49 vs. 0.57 vs. 0.73 vs. 0.21 70.6 vs. 76 vs. 71.4 vs. 112.2 68.3 vs. 70.5 vs. 67.3 vs. 102.7 79.4 vs. 101.2 vs. 83.9 vs 172.7
Jones et al. [44] United States	Dosimetric comparative study (in silico)	n = 10 Medically inoperable early stage EC (*n* = 5 prior EBRT)	IC-BT (HDR) CT	SBRT (HT) PTV (isotropic margin of 2 mm from HR-CTV)	34 Gy in 4 fractions	V150% Uterus D90 boost D90 Uterus D90 PTV D2cc R D2cc S D2cc B	22.7 vs 16.7% *(p* *=* *0.05)* 5.62 vs. 12.10 *(p* *<* *0.005)* 2.76 vs. 8.69 *(p* *<* *0.005)* 2.48 vs. 7.98 *(p* *<* *0.005)* 1.91 vs. 3.01 4.43 vs. 4.68 4.68 vs 4.34
Kauffman et al. [45] United States	Dosimetric comparative study (in silico)	*n* = 6 Medically inoperable early stage EC (n = 3 prior EBRT 46Gy/25–28 fractions)	IC-BT triple-tandem (HDR) MRI + CT	SBRT (3D-Arc and VMAT) PTV (isotropic margin of 5 mm from HR-CTV)	BT alone: 35 Gy in 5 fractions BT + EBRT: 20 Gy in 5 fractions	D2cc R D2cc S D2cc B	*BT* vs. *3D-Arc SBRT* vs. *VMAT-SBRT* 0.40 vs. 0.62 vs. 0.47 0.47 vs. 0.70 vs. 0.37 0.58 vs. 0.83 vs. 0.55
Yildrin et al. [46] Turkey	Dosimetric comparative study (in silico)	*n* = 12 Post-operative early stage EC	IC-BT (HDR) CT	SBRT (VMAT Linac and HT) PTV (isotropic margin of 5 mm from HR-CTV)	25 Gy in 5 fractions	D50 PTV D95 PTV CI HI D2cc R D2cc B	*BT* vs. *Linac SBRT* vs. *HT SBRT* >37.5 vs. 26.15 vs. 25.49 (p < 0.001) >37.5 vs. 25.02 vs 25.11 (p < 0.001) N/A vs. 0.52 vs. 0.29 (p < 0.001) N/A vs. 0.11 vs. 0.06 (*p* = 0003) 24.77 vs. 22 vs. 23.76 (*p* = 0.7 BT vs VMAT, *p* = 0.5 BT vs HT) 19.55 vs. 22.20 vs. 24.21 (p < 0.001)

Dose values are reported as described in the original studies (physical dose or EQ.D2).

### 2.6 Risk of bias assessment

Given the predominance of dosimetric and observational studies, methodological quality was assessed using an adapted approach based on STROBE criteria, focusing on study design, reporting completeness, sample size, outcome reporting, and potential sources of bias. A formal risk of bias tool was not applied due to the heterogeneity of study designs and the dosimetric nature of many included studies. Consensus guidelines were evaluated using the AGREE II.

### 2.7 Data synthesis

A qualitative synthesis of the included studies was performed due to heterogeneity in study design, patient population, treatment planning approaches, and reported outcomes. Results were grouped according to target coverage, OAR exposure, dose distribution and clinical outcomes.

A meta-analysis was not performed because of the limited number of comparable studies and heterogeneity in reported endpoints.

## 3 Results

### 3.1 Systematic review

For this systematic review, eleven articles were selected following the application of stringent inclusion and exclusion criteria, as detailed in the Flow Diagram, in Fig. 1. Eleven studies met the inclusion criteria, comprising nine dosimetric analyses, one retrospective observational cohort, and one expert consensus statement. The majority of studies focused on LACC, with fewer addressing medically inoperable EC or postoperative settings. Considerable heterogeneity was observed in SBRT delivery techniques, fractionation schedules, and planning methodologies. Key characteristics of the included studies are summarized in Table 1.

### 3.2 Survival outcomes in cervical cancer

The only study reporting survival outcomes was the *National Cancer Data Base* (NCDB) analysis by O'Donnell et al. [36], including 15,905 patients with LACC. Most patients received BT (90.5%), whereas SBRT was used in 0.8% of cases. Patients treated with SBRT frequently presented worse baseline characteristics, including higher Charlson/Deyo comorbidity scores (*p* = 0.004).

Unadjusted overall survival (OS) appeared significantly lower with SBRT (30.3 months) than with BT (99.1 months; *p* = 0.001). After propensity-score matching (PSM) for age, stage, comorbidities, and metastases, no statistically significant difference in OS was found between SBRT and BT (HR = 1.477; 95% CI: 0.746–2.926; *p* = 0.263). In contrast, IMRT used as a boost was associated with significantly inferior survival compared with BT (HR = 1.455; 95% CI: 1.300–1.628; *p* < 0.001). Regarding dose delivery, the median biologically effective dose (BED) for SBRT was 75.5 Gy, higher than that recorded for IMRT (63.7 Gy). Dose information for BT was not available in the NCDB, but the authors referenced the expected BT BED range of 86–100 Gy from published literature.

### 3.3 Therapeutic efficacy consensus

The French Society of Radiation Oncology (SFRO) consensus [37] emphasized the indispensable role of BT—particularly combined IC + IS-BT—in delivering curative of 85–90 Gy 2Gy-equivalent dose (EQ.D2) within complex target geometries. The review concluded that SBRT regimens reported in the literature typically deliver <80 Gy EQ.D2 or require significant compromises in OAR protection to achieve BT-like coverage, even when advanced platforms such as CyberKnife (CK) or helical tomotherapy (HT) are used.

### 3.4 Dosimetric comparison between SBRT and BT

#### 3.4.1 Target coverage and dose escalation

The dosimetric evaluation of SBRT vs. BT treatments for LACC yielded divergent results regarding target volume coverage and OAR sparing. Prior to the boost, patients received EBRT, which doses ranged across the reviewed studies from 45 Gy to 50.4 Gy in 25–28 fractions (fr). Benkhaled et al. [38] compared IC + IS-BT (28.4 Gy in 4 fr) with simulated IC-BT plans without interstitial needles and with SBRT plans. Both BT techniques achieved greater intratumoral dose intensification than SBRT, resulting in significantly higher D30% and D50% values. The highest D50% was observed with IC + IS-BT (12.1 Gy/fr), compared with 9.93 Gy/fr for SBRT. Using a comparable CK-based SBRT regimen (28 Gy in 4 fr), Gao et al. [39] also observed superior dose escalation with BT. The cumulative mean D50% was 42.08 Gy for BT vs. 32.94 Gy for margin-free SBRT (*p* < 0.001). Otahal et al. [40], further confirmed higher intratumoral dose escalation with BT 30 Gy in 6 fr, with high-risk clinical target volume (HR-CTV) D30 values of 17.9 Gy/fr, compared with CK-based SBRT (9.4 Gy/fr).

Results for HR-CTV D90 were heterogeneous across studies. Dahbi et al. [41] prescribed 21 Gy in 3 fr and excluded laterally extensive tumors that would require IT-BT. In that setting, BT achieved a significantly higher D90 than SBRT (17 Gy vs. 8.3 Gy; *p* = 0.028). Otahal et al. [40], reported higher HR-CTV D90 values with SBRT than with BT (7.3 Gy/fr vs. 6.1 Gy/fr). However, the mean gross target volume (GTV) D90 favored BT (9.8 Gy/fr) over CK SBRT (8.9 Gy/fr). Conversely, Benkhaled et al. [38] found that margin-free SBRT produced the highest D90 (9.02 Gy/fr), significantly surpassing IC-BT (6.62 Gy/fr) and IC + IS-BT (7.8 Gy/fr) with *p* < 0.001. Likewise, Dincer et al. [42] simulating a 28 Gy in 4 fr boost delivered with stereotactic resonance magnetic-guided adaptive (SMART), achieved a total EQ.D2 D90 (EBRT + boost) that exceeded BT by 5.7 Gy (95.6 Gy vs. 89.7 Gy). However, when applying planning target volume (PTV) margin, D90% decreased to 82.9 Gy EQ.D2. Gao et al. [39] similary showed that SBRT plans outperformed BT in peripheral coverage parameters: HR-CTV achieved higher peripheral coverage (D95: 26.99 Gy and D98 of 25.49 Gy) compared with BT (D95% of 25.89 Gy and D98% of 23.74 Gy; *p* < 0.001). The PTV plan (with a 5 mm margin) also exceeded BT (D95% 27.05 Gy, D98% 25.75 Gy; p < 0.001).

Neumann et al. [43] was the only study in which SBRT was delivered clinically rather than generated virtually. Patients received 30 Gy in 5 fr using two robotic radiosurgery approaches: RRS70 (prescription to the 70% isodose line) and RRS25 (prescription to the 25% isodose line). Both strategies were compared with an individual BT plan (BTRRS) and a reference BT plan (BTref). RRS25—designed to maximize dose intensification—provided the best PTV coverage (V100: 100%), followed by RRS70 (97.1%). Both outperforming BTref (90.9%) and BTRRS (80.6%). RRS25 also achieved the highest HR-CTV D90 (69.5 Gy), whereas BTRRS yielded the lowest value (25.7 Gy).

Two studies evaluated primary EC in medically inoperable patients, treated with radical IC-BT. Jones et al. [44] compared IC-BT (34 Gy in 4 fr to the uterus) with SBRT using HT, which included an integrated boost of 48 Gy and a third volume encompassing uterus and cervix with a 2-mm margin (PTV) up to 12 Gy. SBRT demonstrated significantly higher volumetric coverage: the mean uterine D90 for SBRT with HT was 8.69 Gy/fr compared with 2.76 Gy/fr with IC-BT (*p* < 0.005). SBRT also achieved higher D90 in the boost volume (12.1 vs. 5.62 Gy, p < 0.005) and PTV (7.98 vs. 2.48 Gy, p < 0.005). However, analysis of the high-dose volume (V150%) revealed that BT deposited more very high-dose regions (22.66%) versus SBRT (16.6%). Kauffmann et al. [45] reported a median dose of 35 Gy in 5 fr for IC-BT using triple tandem applicators, compared with SBRT delivered with 3D-Arc and VMAT, no target results were registered.

In contrast, Yildirim et al. [46] evaluated postoperative treatment in patients with EC, receiving adjuvant IC-BT (25 Gy in 5 fr to the vaginal cuff). BT generated hot-spots between 150% and 250% of the prescription dose, while SBRT plans (VMAT and HT) with PTV margin remained between 95% and 107%. However, the limited sample size of this postoperative endometrial cancer subgroup inherently restricts the statistical power and robustness of any derived conclusions, introducing a potential risk of bias that limits its broader contribution to the overall analysis.

#### 3.4.2 Conformity and homogeneity

Gao et al. [39] also demonstrated significantly improved HI and CI with CK-based SBRT plans relative to BT. The mean HI for BT was 4.34, compared with 1.47 for HR-CTV and 1.53 for PTV plan (*p* < 0.001). Mean CI values were 0.75 for BT, 1.12 for HR-CTV, and 1.06 for PTV (p < 0.001). Neumann et al. [43] also showed that SBRT (RRS70 plan) offered superior conformity (0.73) compared with BTref (0.57) and BTRRS (0.49).

#### 3.4.3 OARs sparing

OAR sparing, primarily evaluated by the D2cc, emerged as a key point of differentiation between the modalities.

Three intra-individual dosimetric comparison studies in LACC found that SBRT delivered significantly lower doses to OARs. Otahal et al. [40] concluded that the SBRT CK was statistically superior in OAR sparing, with doses 20–30% lower to the volumes of the bladder, rectum, and sigmoid compared to BT (*p* < 0.05). Specifically, the mean D2ccR was 1.8 Gy/fr with CK vs. 3.0 Gy/fr with BT. Benkhaled et al. [38] showed that SBRT delivered the lowest D2cc to all critical OARs. The D2ccR was 2.71 Gy/fr with SBRT, significantly lower than 3.58 Gy/fr for IC + IS−BT (*p* < 0.001). D2ccB was 4.99 Gy/fr with SBRT, lower than 5.49 Gy/fr with IC + IS−BT and 6.46 Gy/fr with IC − BT (p < 0.001). Notably, the addition of interstitial needles reduced D2ccB by approximately 1 Gy/fr compared with IC − BT alone; however, SBRT still achieved lower values overall. Gao et al. [39] demonstrated that the HR-CTV plan was significantly superior to BT, also reducing the accumulated D2ccR (15.84 Gy vs. 17.36 Gy, *p* = 0.019) and D2ccB (18.45 Gy vs. 19.64 Gy, *p* = 0.007). The radiobiological analysis of the normal tissue complication probability (NTCP) for the rectum was significantly lower with HR − CTV SBRT plan (9.50%) compared to BT (28.08%, *p* < 0.001), indicating a substantially lower predicted risk of toxicity. The SBRT advantage in OAR sparing is highly sensitive to the planning strategy. Gao et al. [39] highlighted this by showing that the inclusion of a 5 mm PTV margin resulted in a substantial increase dose to OARs (D2ccR 21.92 Gy, D2ccS 20 Gy and D2ccB22.48 Gy; p < 0.001).

In contrast, Dahbi et al. [41] found better OAR protection with BT. Total bladder EQ.D2 was significantly lower with BT than with SBRT (74.6 vs. 84.7 Gy; *p* = 0.019). Similar findings were observed for the sigmoid colon (67.9 vs. 75.75 Gy; *p* = 0.0173). Rectal doses were comparable between both techniques (71.8 vs. 74.6 Gy; *p* = 0.256). Dincer et al. [42] reported slightly lower bladder doses with SBRT than with BT (81.4 vs. 86.4 Gy). However, sigmoid doses were higher with SBRT (73.6 vs. 65.7 Gy). In the study by Neumann et al. [43], rectal exposure was similar between techniques, although bladder doses favored BT (79.4 vs. 83.9 Gy). Importantly, the highly intensified RRS25 strategy exceeded OAR tolerance limits, reaching a bladder D2cc of 172.7 Gy and therefore was not considered clinically feasible.

OAR sparing remains a key factor in other gynecologic sites. Jones et al. [44] found no significant difference between definitive treatment for EC in the mean bladder dose (D2ccB: 4.43 Gy/fr for SBRT vs. 4.68 Gy/fr for BT). However, SBRT significantly increased the dose to the rectum and sigmoid compared to BT (D2ccR: 3.07 Gy/fr with SBRT vs. 1.91 Gy/fr with BT; D2ccS 4.48 Gy/fr vs. 4.05 Gy/fr). Kauffmann et al. [45] reported significantly greater OAR preservation with triple tandem BT planning compared to 3D-CRT SBRT, although VMAT SBRT yielded similar D2cc results to BT. Yildrim et al. [46] found that adjuvant BT to vaginal vault offered a significant advantage over VMAT and HT in bladder dose (D2ccB: 19.55 Gy with BT vs. 22.20 Gy with VMAT vs. 24.21 Gy with HT, *p* < 0.001).

## 4 Discussion

This systematic review synthesizes the current evidence comparing SBRT and BT for non-metastatic gynecologic malignancies across dosimetric, technical, and clinical endpoints.

### 4.1 Target delineation, motion uncertainty, and treatment geometry

One of the most fundamental distinctions between the two modalities lies in target geometry and motion management. SBRT necessarily requires a PTV margin to compensate for interfraction and intrafraction organ motion. Cervical excursion has been reported to average 3 mm but may reach 18 mm in extreme cases, driven by bladder filling, rectal distension, and uterine mobility [37], [47]. Even with image guidance, reproducing internal anatomy with the precision required for ablative dosing remains difficult [48]. In contrast, BT minimizes geometric uncertainty by placing the source directly inside or adjacent to the tumor [37]. This anatomical proximity eliminates the need for PTV margins and enables a level of geometric fidelity unobtainable with external-beam techniques [49]. The absence of margins is not simply a dosimetric convenience but a biological necessity: high-dose regions are deliberately concentrated in the central tumor core, where hypoxic and radioresistant subclones predominate [37], [38]. A notable finding of this review is that only two studies for LACC—Gao et al. [39] and Dincer et al. [42]—explicitly incorporated a PTV margin in SBRT planning for cervical cancer, despite the fact that margin expansion is mandatory in any external-beam technique treating a mobile pelvic target. This methodological detail is critical: both studies showed a marked loss of dosimetric advantage once realistic margins were applied. Gao et al. [39] demonstrated that PTV expansion substantially increased rectal and bladder doses, and only tumors with HR-CTV < 56.5 cm^3^ maintained toxicity levels comparable to BT. Dincer et al. [42] reported similar observations, noting that even small margins decreased SBRT's target volume benefits over BT. Taken together, these findings underscore that SBRT performance is margin-dependent, and that most published comparisons, which omit margins, likely overestimate its clinical feasibility as a substitute when BT is available.

### 4.2 Dose escalation, hot-spot dynamics, and the radiobiological advantage of BT

Dose escalation remains the principal domain where BT shows a clear and consistent advantage over SBRT. Across the dosimetric comparisons included in this review, BT provided substantially higher intratumoral dose heterogeneity and superior high-dose gradients, both of which are central to its biological effectiveness [50].

Although SBRT generally achieved competitive coverage metrics, particularly for peripheral parameters such as D90, D95 or the conformity index (CI), these advantages did not translate into equivalent dose escalation within the central tumor region. In several studies, SBRT achieved higher or comparable D90 values relative to BT, especially in plans generated without PTV margins [38], [39], [40], [43]. However, the apparent superiority of SBRT in D90 was often offset by its relative deficit in high-dose subvolumes, where BT consistently outperformed all SBRT techniques. Specifically, multiple studies showed that BT delivered markedly higher D30 and D50, indicating a concentration of dose in the tumor core that SBRT could not emulate even with optimized inverse planning. These high-dose regions, frequently exceeding 150–200% of the prescription, represent a defining physical characteristic of BT. In contrast, SBRT dose distributions remained inherently more homogeneous, with steep dose gradients achievable only outside, not within, the target volume [39]. Even when SBRT achieved adequate D90, its lower D30 and D50 values reflect the inability of external-beam techniques to recreate the steep, localized hotspots produced by IC/IS-BT [38], [39], [40]. Margin application also limited any attempt to increase central hot spots [39], [42].

Overall, these findings indicate that while SBRT can provide competitive peripheral coverage and occasionally favorable D90 under idealized no-margin conditions, it does not replicate the high-dose gradients or central dose escalation characteristic of BT. These conclusions are consistent with the review conducted by the AIRO Gynaecology Study Group, which emphasized that external-beam boost techniques, including stereotactic approaches, are currently unable to fully reproduce the geometric and radiobiological advantages of BT [51].

### 4.3 Clinical outcomes

Only one population-based study provided survival data, derived from the NCDB in which SBRT was used in a very small proportion of patients and predominantly in those with more advanced disease or significant comorbidities [39], Although OS appeared inferior for SBRT in unadjusted analyses, this difference was no longer statistically significant after adjustment for prognostic factors, suggesting that treatment selection bias played a major role. It should be emphasized that SBRT patients represented only 0.8% of the cohort, limiting statistical power and SBRT was more frequently used in patients with worse performance status and higher disease burden. Importantly, the same analysis demonstrated that SBRT achieved a higher BED than IMRT boost, while IMRT was associated with significantly worse survival, reinforcing the concept that SBRT may represent a more appropriate external-beam alternative when BT cannot be delivered. Nevertheless, a significant source of bias in these clinical data arises from the overwhelming predominance of a single database cohort, wherein approximately 90.5% of the overall population underwent BT. This marked imbalance inherently favors BT in the comparative survival analysis. It is crucial to note that SBRT retains substantial potential for further technological refinement and clinical validation, particularly given that the current survival data remain immature and heavily influenced by patient-selection factors.

Regarding treatment-related toxicities, the clinical evaluation of acute and late adverse events remains a critical factor for patient counseling. Prospective phase I/II trials of SBRT boosts have reported acceptable acute gastrointestinal and genitourinary toxicities, but concerns persist regarding late severe complications—such as fistulas or severe necrosis—when external doses mimic BT distributions without real-time tracking [26], [27], [28], [29], [30], [31], [32]. A comprehensive, prospective evaluation of both acute and late toxicities for both modalities is highly warranted to guide clinicians in treatment selection.

### 4.4 Patient-reported outcomes, quality of life, and treatment burden

An important aspect that remains insufficiently addressed in the current literature is the impact of treatment modality on patient-reported outcomes (PROs) and quality of life. Most studies included in this review were dosimetric analyses and did not report patient-centered outcomes, such as treatment-related symptoms, physical functioning, psychological distress or sexual health.

From a practical perspective, SBRT offers several potential advantages. Unlike BT, it is completely non-invasive and does not require applicator or interstitial needles insertion, anesthesia, operating-room resources, or hospitalization. Consequently, SBRT may reduce procedural discomfort and treatment-related anxiety for selected patients. These factors could potentially improve treatment acceptability and patient experience, particularly in elderly individuals, patients with significant comorbidities, or those unable to undergo invasive procedures. However, the absence of prospective comparative studies prevents any definitive conclusions regarding quality of life or patient preference.

Consequently, no evidence-based comparison of quality of life between BT and SBRT can currently be established.

### 4.5 Accessibility and health-system considerations

Accessibility remains a major factor influencing the potential role of SBRT as an alternative to BT. Although BT is considered the standard of care for dose escalation in gynecologic cancers, its implementation requires specialized equipment, dedicated facilities, and experienced multidisciplinary teams. As a result, access to BT varies considerably across institutions and healthcare systems, and declining utilization has been reported in several countries. The AIRO Interventional Radiotherapy Study Group has highlighted that the progressive reduction in BT utilization represents an important challenge for modern radiation oncology and stressed the need to preserve expertise, training programs, and dedicated infrastructures to ensure adequate patient access to this treatment modality [52].

SBRT may represent a practical alternative in centers where BT expertise or infrastructure is unavailable. Modern linear accelerators and stereotactic platforms are more widely distributed than dedicated BT services in some healthcare settings, potentially facilitating treatment access. In addition, SBRT avoids anesthesia and invasive procedures, which may reduce certain procedural costs and logistical barriers.

Nevertheless, the comparative cost-effectiveness of SBRT and BT remains poorly studied. Any potential economic advantages of SBRT must be weighed against the possibility of inferior dose escalation and the lack of robust long-term clinical outcome data. Formal health-economic analyses are needed to determine the relative value of both approaches across different healthcare systems.

### 4.6 Specific considerations for endometrial Cancer

In EC, the evidence base is even more limited. The few comparative studies included in this review evaluated SBRT primarily as an alternative for medically inoperable patients or in postoperative settings, and all three incorporated a PTV margin to account for uterine and vaginal motion, thereby reflecting clinically realistic treatment conditions [44], [45], [46]. BT consistently achieved higher intratumoral dose escalation while limiting unnecessary irradiation of surrounding tissues in postoperative scenario [46]. In contrast, Jones et al. [44] showed demonstrated higher doses to D90 volumes with SBRT for inoperable EC.

These findings support that SBRT could be reserved for selected patients in whom anesthesia, applicator placement, or procedural risks preclude standard BT.

### 4.7 Methodological limitations and strengths

This review is primarily limited by the nature of the available evidence. Most included studies were dosimetric in silico analyses, which, although informative regarding target coverage and organ-at-risk exposure, cannot be directly extrapolated to clinical outcomes. Considerable heterogeneity existed across studies in terms of clinical scenarios, target delineation, dose prescription, fractionation, imaging modality, and treatment platform. Variability in BT techniques, particularly the inclusion or omission of interstitial components, further complicates direct comparisons.

A major methodological limitation is that only two studies incorporated a PTV margin in SBRT planning for cervical cancer, despite well-documented pelvic organ motion. Comparisons omitting margins likely overestimate SBRT's dosimetric performance and underestimate OAR doses. Studies that included margins consistently showed reduced target coverage and loss of OAR sparing, highlighting the importance of realistic motion modeling.

Beyond methodological heterogeneity, an important limitation relates to the absence of high-level clinical evidence. While dosimetric comparisons provide valuable mechanistic insights, they do not replace the need for direct clinical studies evaluating real patient outcomes. To date, no randomized phase III clinical trial has directly compared SBRT and BT in non-metastatic gynecologic cancers. Consequently, it is not possible to reliably compare acute toxicity, late adverse effects, quality of life, local control, or overall survival between both modalities based on current evidence.

The single population-based survival analysis included in this review was retrospective and subject to significant selection bias, with SBRT preferentially administered to patients with less favorable baseline characteristics. Additionally, the overall evidence base was heavily influenced by a single large retrospective cohort, which accounted for the vast majority of included patients and may have disproportionately affected the interpretation of clinical outcomes. Moreover, the lack of detailed information regarding BT dose and fractionation in large registries restricts interpretability and precludes robust comparative effectiveness conclusions.

Despite these limitations, this review has several strengths. It provides a focused and contemporary synthesis of the evidence comparing SBRT and BT in gynecologic malignancies, with particular emphasis on dose escalation and target geometry. Importantly, many of the included SBRT studies were performed using state-of-the-art technologies, including robotic radiosurgery platforms, HT, VMAT and SMART. These advanced systems represent significant technological innovation and reflect high-quality treatment environments, enhancing precision, adaptive capabilities, and image guidance. By incorporating data from such modern platforms, this review captures the current technological ceiling of SBRT performance rather than outdated external-beam techniques.

In addition, separating cervical and endometrial cancer analyses improves interpretability, and the emphasis on dosimetric endpoints ensures internal consistency across studies in a field where prospective clinical data remain scarce. The structured evaluation of margin-dependent planning further strengthens the relevance of this review for contemporary clinical decision-making.

### 4.8 Clinical implications and future directions

The evidence supports BT as the optimal modality for dose escalation in gynecologic cancers, owing to its ability to deliver highly heterogeneous, ultra-high intratumoral doses without the need for PTV margins. This position is consistent with recommendations from the AIRO Gynaecology Study Group, which continue to recognize BT as the preferred boost modality for LACC cancer whenever feasible [51]. SBRT may serve as a selective alternative in patients who are not candidates for BT, and appears preferable to IMRT as a boost in this context. However, efforts to improve access to BT services and maintain specialized expertise remain essential, as emphasized by the AIRO Interventional Radiotherapy Study Group [52]. Future studies should focus on prospective phase II/III trials comparing BT and SBRT with mandatory PTV margins, standardized reporting, incorporation of patient-reported outcomes, and advanced motion management strategies.

## 5 Conclusion

BT remains the reference standard for dose escalation in non-metastatic gynecologic cancers, particularly in locally advanced cervical cancer, due to its superior intratumoral dose heterogeneity and central dose delivery. Contemporary SBRT demonstrates competitive peripheral target coverage and high conformity, representing the most technically advanced external-beam alternative when BT is not feasible.

Given the scarcity of clinical evidence and the absence of randomized comparisons, SBRT should be considered a technically promising but investigational complementary strategy rather than a replacement for BT. When BT is unavailable or contraindicated, SBRT may represent a reasonable alternative in carefully selected patients, although it cannot currently be considered equivalent to BT. Future prospective studies with standardized planning, mandatory motion management, and long-term clinical outcomes are required to determine whether dosimetric advantages translate into meaningful clinical benefit.

## Declaration of generative AI

During the preparation of this work the authors used AI-assisted tools to improve language clarity and organization of the manuscript. The authors reviewed and edited all content and take full responsibility for the final version of the manuscript.

## CRediT authorship contribution statement

**Paula Vicente Ruiz:** Conceptualization, Methodology, Writing – original draft. **Ana Illescas Vacas:** Conceptualization, Methodology. **Ángel Vilches Arenas:** Conceptualization, Methodology, Writing – review & editing, Supervision.

## Funding

This research did not receive any specific grant from funding agencies in the public, commercial, or not-for-profit sectors.

## Declaration of competing interest

The authors declare no competing financial interests or personal relationships that could have appeared to influence the work reported in this paper.
